# Chronologic versus Biologic Aging of the Human Choroid

**DOI:** 10.1155/2013/378206

**Published:** 2013-12-25

**Authors:** Christian Albrecht May

**Affiliations:** Department of Anatomy, Medical Faculty Carl Gustav Carus, TU Dresden, Fetscherstra**β**e 74, 01307 Dresden, Germany

## Abstract

Several aspects of chronologic and biologic aging in the human choroid are reviewed from the literature. They often reveal methodological problems for age-dependent changes of the following parameters: choroidal thickness, choroidal pigmentation, choroidal vasculature and blood flow, and choroidal innervation. On reinterpreting some data of studies concerning Bruch's membrane, changes observed at different age points seem more likely to be nonlinear. Concluding from the data presented so far, chronologic aging should not be used as a factor for physiological changes in the human choroid. Longitudinal study designs are necessary to further establish the impact of age. Meanwhile, a more biologic oriented model of aging processes in the choroid should be established, including specified conditions (e.g., light exposure and refractory state). This would help to define more individual strategies for prevention and early stages of a certain defined disease.

## 1. Introduction 

Aging in our cultural setting is a complex of factors leading to disadvantages and potential diseases. Aging as a complex development leading to more knowledge and potential wisdom is widely neglected in the natural sciences. Aging is potentially dangerous and should be prevented if possible—but what does aging in a specific setting mean?

Even though ocular physiology has its primary focus on retinal light perception and transparency of the optic apparatus, a number of functions are known to be located in the choroid including nutrition of the outer retina, fine-tuning of the central fovea (short term during accommodation and long term during eye growth), and buffering of the intraocular temperature. All these conditions are crucial for proper vision and should remain functional throughout life. The present review focuses on the impact of chronologic and biologic aspects on the function of the choroid analysing the published literature. The key questions should clarify which aspects concerning aging are known at present in the human choroid and how can they lead our understanding of this process.

## 2. Choroidal Thickness

### 2.1. Data

Thinning of the total choroid over age was described in several cross-sectional studies. Morphological postmortem observations revealed a decrease up to 57% ([1] 95 donors, age range 6–100 years; [2] 45 donors, age range 17–84 years). The age-related thinning was also observed using optic coherence tomography in vivo ([3] 43 volunteers, age range 23–88 years; [4] 34 volunteers, age range 22–78 years; [5] 3468 volunteers, age range 50–93 years). One study, however, observed an age-related thinning effect only in myopic eyes ([6] 32 volunteers, age range 19–80 years). Another study revealed differences in choroidal thickness between adult (up to 60 years) and senescent (over 60 years) healthy volunteers (*n* = 210) without a constant age-dependent correlation [7]. No correlation of age and thinning of the human choroid was described more recently ([8] 36 volunteers, age range 28–79 years; [9] 45 volunteers, age range 23–80 years).

### 2.2. Discussion

For a long time, supported by numerous studies, thinning of the choroid was described as a chronologic aging process. Unfortunately, all studies had a cross-sectional design and they did not differ between subgroups of healthy volunteers (like gender, general health conditions, vision development, or ocular activity). Therefore, the relation to chronologic aging is not as yet verified. A longitudinal study design and a subgroup analysis are necessary for further studies covering this topic.

## 3. Choroidal Pigmentation

### 3.1. Data

In vivo, the pigmentation of the posterior eye fundus is a combination of the retinal pigment epithelium (RPE) and the choroidal melanocytes. Fluorescence optical measurements on paraffin sections of 38 donors (aged 2 to 90 years; 19 whites, 16 American blacks) showed a trend of decreasing content with aging in both RPE and choroidal melanocytes [10]. By contrast, biochemical measurements of melanin in the peripheral and macular choroid (11 donors, aged 17–88 years) revealed two-to-threefold higher levels in the macula region compared to the more peripheral regions, but no alteration with aging [11].

### 3.2. Discussion

It remains to be determined whether melanin decreases physiologically with age. Its importance for normal eye function has been acknowledged for both RPE [12] and choroid [13], and a decrease of melanin has been described in numerous pathologic conditions.

## 4. Choroidal Vasculature and Blood Flow

### 4.1. Data

Early studies on age-related changes of the choroidal vasculature focused on the choriocapillaris and observed a morphologic reduction in the cross-sectional area ([14] 5 donors, ages 3,5 months, 40 and 99 years; Ring and Fujino [15], 125 donors, age range 22nd week of gestation–104 years; Ramrattan et al. [1], 95 donors, age range 6–100 years; Rymgayłło-Jankowska et al. [2], 45 donors, age range 17–84 years). Physiologic blood flow measurements suggested a “linear” reduction of choroidal blood flow ([16] 29 volunteers, age range 15–76 years; [17] 130 volunteers, age range 19–83 years; [18] 118 volunteers, age range 19–75 years) and a reduction of choroidal arterioles and of the capillary filling time in adults over 50 years of age ([19] 30 volunteers, age range 21–81). A gender-biased analysis of choroidal blood flow [20] revealed significant lower levels in older (around 60 years of age; *n* = 11) versus younger females (around 30 years of age; *n* = 16) but not in males (same age groups; *n* = 26).

The endothelial cells of the choroidal vessels showed no age-related changes regarding their cytoarchitecture ([21] 65 donors, age range 7–87 years).

### 4.2. Discussion

As pointed out already for the choroidal thickness, most of the older studies had a cross-sectional design and did not differ among subgroups of healthy volunteers. Therefore, the relation of chronologic aging and vascular changes (function: reduced blood flow; structure: reduced choriocapillary density) is not as yet verified. Again, a longitudinal study design and a subgroup analysis are necessary for further studies covering this topic. New techniques [22] are on their way to make such studies possible.

## 5. Choroidal Innervation and Other Single Aspects of Choroidal Tissue

### 5.1. Data

The complex innervation of the choroid was described using tissue samples of all age groups. The number of neurons within the choroid and the pattern of different neurotransmitters did not change with age ([23] 32 donors, age range 12–95 years). However, only two papers specifically addressed age in their analysis: one described a decrease of adrenergic fibres with less varicosities in four donors aged 70–75 years compared to four patients aged 40–45 years [24]. The other paper investigated the VIP positive nerve fibres in the submacular region of 35 donors between 21 and 93 years of age: within a high interindividual range a statistical decrease could be calculated [25].

A decrease of hyaluronic acid in the choroid stroma was observed, reaching almost complete absence in donors over 50 years of age ([26] 11 donors, age range 28–94 years).

The nonvascular smooth muscle cells in the choroid showed no age-related changes ([27] 19 donors, age range fetal to 94 years).

### 5.2. Discussion

Single descriptions suggest nerve fibre changes in the aging choroid and in the extracellular composition; these data have to be confirmed.

## 6. Bruch's Membrane

### 6.1. Data

The most widely documented age-related changes of the choroid are in human Bruch's membrane and include thickening and changes in their composition (for reviews see [28, 29]).

The thickening of Bruch's membrane seemed to start in the periphery; in the macular region first thickening was observed from 45 years of age ([30] 31 donors, age range 12 days to 80 years). By some contrast, other groups proposed a linear correlation with age ([1] 95 donors, age range 6–100 years; [31] 88 donors, age range 1–98 years; [2] 45 donors, age range 17–84 years).

Morphological aspects which might represent (at least partly) the thickening of Bruch's membrane include an accumulation of lipids, mainly cholesterol ([32, 33] 20 donors, age range 17–92 years; [34] 4 donors, aged 27, 41, 76, and 78 years), which seems, however, to persist after the age of 60 years ([35] 27 donors, age range 60–95 years). The amount of type III collagen remained constant during life as did the amount of enzymatically formed collagen cross-links, while noncollagen proteins increased significantly ([36] 9 donors, age range 1–78 years). This is in some contrast to the observations of Newsome, who reported an increase of collagen and elastin in the macula region [30].

Further changes in Bruch's membrane include accumulation of glycoconjugates and glycosaminoglycans [30], accumulation of degraded gelatinase (mmp2 and mmp9; [37] 32 donors, age range 17–82 years; [38] 29 donors, age range 21–99 years; [39] 10 donors, age range 21–84 years), timp-3 ([40] 36 donors, age range 14–96 years), pentosidine ([41] 2 donors, 20 months and 82 years of age), AGEs ([42] 8 donors, age range 34–89 years), and nitrotyrosine [43]. In addition, there was a loss of hyaluronic acid with no staining in tissues over 50 years of age ([26] age range 28–94 years).

All mentioned factors might contribute to the decline in the solubility of Bruch's membrane ([36] 76 eyes, age range 1–92 years) and to the loss of elasticity ([44] 13 donors, age range 21–97 years). Other functional tests showed decreased diffusion for water (exponential relation; Fisher [45], 12 donor eyes, age range 22–71 years; Moore et al. [46], 13 donors, age range 17–90 years; Starita et al. [47], 12 donor eyes, age range 17–91 years; Starita et al. [48], 26 donors, age range 1–91 years), serum proteins ([49] 17 donors, age range 9–85 years), amino acids ([50] 19 donors, age range 13–89 years), taurine ([51] 29 donors, age range 13–88 years), and macromolecular proteins ([52] 14 donors, age range 4–92 years).

It was shown that the proposed age-related changes of Bruch's membrane influenced the gene expression profile of the RPE ([53] 10 donors, age range 31–81 years).

Within Bruch's membrane, the appearance of drusen is often seen in adult eyes. Although a correlation between soft drusen but not hard drusen and increase with age was documented ([54] 23 donors, age range 36–94 years), only hard drusen are nowadays considered to be a consequence of normal aging [29]. Randomized screenings observed in eyes of donors older than 40 years a presence of hard drusen in at least 50% of the donors; one study noted no clear age correlation ([55] 202 donors, age range 43–96 years), and the other one noted a tendency of increase with age ([56] 46 donors, age range 42–95 years). A longitudinal study showed that drusen do not necessarily persist over time but show reversibility of over 30% ([57] 483 volunteers, age range at beginning not specified, follow-up after 5 years).

### 6.2. Discussion

While considering chronologic aging as a factor for changes in Bruch's membrane two incisions seem to take place: one is around forty and the other one around sixty. In reevaluating some data proposing linear changes over the whole age range, it becomes apparent that between 40 and 60 years of age some parameters like the diffusion of macromolecules (e.g., [52]) rather show a steady state than a linear constant decrease. Assuming that there is no complete linear chronologic aging in Bruch's membrane, three characteristic periods could be hypothesized. The first period (up to 40 years of age) is characterized by a peripheral thickening of Bruch's membrane caused by accumulation of lipids, changes in the enzymatic profile of extracellular material regulators, and a subsequent decrease of Bruch's membrane solubility and diffusion. The second period (between 40 and 60 years of age) is characterized by thickening of the macular Bruch's membrane, further accumulation of lipids, a more frequent presence of drusen, but some lingering of Bruch's membrane permeability. The third period (older than 60 years of age) shows no further lipid accumulation but further increase of drusen and decrease of permeability. This last period shows the greatest variability making it difficult to establish age-related aspects in this subgroup.

Even with the greater number of studies, a longitudinal study design is almost not present. Only the Waterman study reported a follow-up of choroidal parameters after 5 years proofing that the morphological appearance of drusen is time sensitive; a clear increase of the number of drusen could not be demonstrated within the five-year period. It is still important to remember that even profound morphological changes are reversible to some extent.

Summarizing the discussions of the different aspects of chronologic age on changes of the choroid mentioned above, it becomes apparent that chronologic aging might not be helpful to understand the “normal” changes and variations of the choroid during time.

Having these difficulties in mind, a more biologic aging was suggested by Sarks characterizing different stages between normal and pathologic conditions. In this study, donor eyes were grouped according to their appearance of Bruch's membrane ([58] 216 donors, age range 43–97 years, 6 groups). Although there was some correlation with chronologic age, individual factors of aging were considered as being more important. A second study followed the rules of Sarks ([59] comparing the data of Sarks with a Japanese population) but it did not became a standard for further age-related aspects. The problem with such a grouping is the implicit idea that aging is a prestage of disease and therefore necessary to prevent.

In terms of nonpathological changes of the choroid during life we might follow the ideas of Gerontologist Aubrey Nicholas Jasper De Grey, who pointed to the body's basal activity of metabolism leading to some kind of useless end products [60]. Accumulation over time might finally lead to pathological conditions or diseases, depending on the body's capability to deal with these often toxic end products. However, if the body changes its strategies or supply, these accumulations are reversible as is aging to a certain degree. De Grey's seven damaging events and their transduction to the choroid are as follows.

(1)* Tissue Stiffening*. Two functional aspects lead to the aspects of stability of the choroid—one is the sponge-like character due to the intense vascularisation allowing variation of the total thickness and the other is the elastic counterforce during accommodation. The first aspect is structurally realized by a loosely arranged network of collagen fibres and nonvascular smooth muscle cells. This dynamic system seems to remain fairly constant during chronologic aging [27, 61], while collagen thickening and changes in the density and arrangement of the smooth muscle cells can be observed in nonhealthy conditions (e.g., own unpublished observations in glaucomatous eyes). There is a high interindividual variation of a certain subgroup of nonvascular smooth muscle cells around the macular region [61] which has not yet been assigned to certain conditions. The second aspect is supported by elastic fibres within Bruch's membrane and the stroma of the choroid (reviewed by [62]). Since elastic fibres cannot be replaced during life, they are structures undergoing irreversible changes with age (e.g., calcification of Bruch's membrane elastic fibres; reviewed by [63]). Interestingly, measurements of the elasticity of the choroid showed only mild or no correlation with chronologic aging ([64, 65] 8 donors, age range 21–79 years; [44] 13 donors, age range 21–97 years; [66] 24 donors, age range 30–74 years); therefore, some elastic properties not yet specified seem to remain beside the elastic fibres.

(2) *Extracellular Debris*. Although the choroid is a highly vascularized tissue, its main capillary bed is the choriocapillaris showing strong interaction to Bruch's membrane and the RPE. In addition, the choroid is specific in having no true lymphatic drainage [67]. To cope with this fact, older eyes show numerous debris in the inner sclera which might act as a trash can for the choroid in that respect and explain the loss of scleral permeability with age [68]. Accumulation of extracellular debris towards Bruch's membrane also occurs as a general thickening and as the described Drusen formation, which seems to be a temporary effect at the first place [57], showing also some genetic predispositions [69]. With these two borders absorbing and accumulating extracellular debris, the choroid itself remains balanced over a long time concerning its extracellular composition. A completely unknown area is the influence of extraocular conditions like nutrition on debris formation: first loose observations were published for lipids [35] and in an animal model for Zinc [70], but much more effort should be made in this respect.

(3) *Intracellular Debris and* (4) *Mitochondrial Defects*. Almost no data exists about intracellular changes of choroidal cells, which include fibrocytes/fibroblasts, melanocytes, immune cells (mainly macrophages and mast cells), and vascular cells (endothelial cells, pericytes, and smooth muscle cells). Melanocytes might change their type of melanin as seen in cell culture experiments [71] and as reviewed for melanocytes in general [72, 73]. Macrophages can change their state of activity as seen in early pathology [74]. Mast cells show diversity in the human uvea [75] and some general age-related characteristics (reviewed by [76]). Specific studies for the human choroid do not exist. In contrast, changes in the adjacent human RPE are broadly documented.

(5) *Cell Overproliferation*. Since the inner eye has a very strict capacity, proliferation as a tool to cope with metabolic imbalance is negligible. However, looking at conditions considered as pathologic, fibroblasts are able to induce proliferation of endothelial cells and neovascularisation ([77–79]). This activity is restricted towards Bruch's membrane and the RPE.

(6) *Cell Loss and* (7) *Cell Death Resistance*. Similar to intracellular changes, almost no data exists about reduced cell densities within the human choroid or the strategies of cells in the choroid to avoid cell death.

In this careful observation of the data concerning age and changes in the choroid it becomes evident that quite a number of factors are hypothesised, but not sufficiently demonstrated yet. To preserve metabolic activity and function, the choroid develops a number of strategies which were touched above but need much more research to establish. Although a number of changes occur frequently correlated with age in the populations studied, we should beware of restricting this data to a linear, irreversible cascade of events leading to pathological conditions. Longitudinal study designs are necessary to further establish the impact of age. Meanwhile, age should not be propagated as an unswayable factor for diseases or predisease conditions. This would help to define more individual strategies for prevention and early stages of a certain defined disease.

## References

[1] Ramrattan RS, Van der Schaft TL, Mooy CM, De Bruijn WC, Mulder PG, De Jong PT (1994). Morphometric analysis of Bruch’s membrane, the choriocapillaris, and the choroid in aging. *Investigative Ophthalmology & Visual Science*.

[2] Rymgayłło-Jankowska B, Szczesny P, Zagórski Z (2002). Morphological analysis of age-related changes in the human choroid. *Klinika Oczna*.

[3] Ikuno Y, Kawaguchi K, Nouchi T, Yasuno Y (2010). Choroidal thickness in healthy Japanese subjects. *Investigative Ophthalmology & Visual Science*.

[4] Manjunath V, Taha M, Fujimoto JG, Duker JS (2010). Choroidal thickness in normal eyes measured using cirrus HD optical coherence tomography. *The American Journal of Ophthalmology*.

[5] Wei WB, Xu L, Jonas JB (2013). Subfoveal choroidal thickness: the Beijing Eye Study. *Ophthalmology*.

[6] Esmaeelpour M, Považay B, Hermann B (2010). Three-dimensional 1060-nm OCT: choroidal thickness maps in normal subjects and improved posterior segment visualization in cataract patients. *Investigative Ophthalmology & Visual Science*.

[7] Ding X, Li J, Zeng J (2011). Choroidal thickness in healthy Chinese subjects. *Investigative Ophthalmology & Visual Science*.

[8] Ho J, Branchini L, Regatieri C, Krishnan C, Fujimoto JG, Duker JS (2011). Analysis of normal peripapillary choroidal thickness via spectral domain optical coherence tomography. *Ophthalmology*.

[9] Shin JW, Shin YU, Cho HY, Lee BR (2012). Measurement of choroidal thickness in normal eyes using 3D OCT-1000 spectral domain optical coherence tomography. *Korean Journal of Ophthalmology*.

[10] Weiter JJ, Delori FC, Wing GL, Fitch KA (1986). Retinal pigment epithelial lipofuscin and melanin and choroidal melanin in human eyes. *Investigative Ophthalmology & Visual Science*.

[11] Hayasaka S (1989). Aging changes in lipofuscin, lysosomes and melanin in the macular area of human retina and choroid. *Japanese Journal of Ophthalmology*.

[12] Schraermeyer U, Heimann K (1999). Current understanding on the role of retinal pigment epithelium and its pigmentation. *Pigment Cell Research*.

[13] Peters S, Lamah T, Kokkinou D, Bartz-Schmidt K-U, Schraermeyer U (2006). Melanin protects choroidal blood vessels against light toxicity. *Zeitschrift für Naturforschung C*.

[14] Klien BA (1966). Regional and aging characteristics of the normal choriocapillaris in flat preparations. preliminary remarks. *The American Journal of Ophthalmology*.

[15] Ring HG, Fujino T (1967). Observations on the anatomy and pathology of the choroidal vasculature. *Archives of Ophthalmology*.

[16] Grunwald JE, Hariprasad SM, DuPont J (1998). Effect of aging on foveolar choroidal circulation. *Archives of Ophthalmology*.

[17] Dallinger S, Findl O, Strenn K, Eichler H-G, Wolzt M, Schmetterer L (1998). Age dependence of choroidal blood flow. *Journal of the American Geriatrics Society*.

[18] Lam AK, Chan S-T, Chan H, Chan B (2003). The effect of age on ocular blood supply determined by pulsatile ocular blood flow and color Doppler ultrasonography. *Optometry & Vision Science*.

[19] Ito YN, Mori K, Young-Duvall J, Yoneya S (2001). Aging changes of the choroidal dye filling pattern in indocyanine green angiography of normal subjects. *Retina*.

[20] Kavroulaki D, Gugleta K, Kochkorov A, Katamay R, Flammer J, Orgul S (2010). Influence of gender and menopausal status on peripheral and choroidal circulation. *Acta Ophthalmologica*.

[21] Guymer RH, Bird AC, Hageman GS (2004). Cytoarchitecture of choroidal capillary endothelial cells. *Investigative Ophthalmology & Visual Science*.

[22] Kim DY, Fingler J, Zawadzki RJ (2013). Optical imaging of the chorioretinal vasculature in the living human eye. *Proceedings of the National Academy of Sciences of the United States of America*.

[23] May CA, Neuhuber W, Lütjen-Drecoll E (2004). Immunohistochemical classification and functional morphology of human choroidal ganglion cells. *Investigative Ophthalmology & Visual Science*.

[24] Nuzzi R, Finazzo C, Grignolo FM (1996). Changes in adrenergic innervation of the choroid during aging. *Journal Francais D’Ophtalmologie*.

[25] Jablonski MM, Iannaccone A, Reynolds DH (2007). Age-related decline in VIP-positive parasympathetic nerve fibers in the human submacular choroid. *Investigative Ophthalmology & Visual Science*.

[26] Tate DJ, Oliver PD, Miceli MV, Stern R, Shuster S, Newsome DA (1993). Age-dependent change in the hyaluronic acid content of the human chorioretinal complex. *Archives of Ophthalmology*.

[27] Poukens V, Glasgow BJ, Demer JL (1998). Nonvascular contractile cells in sciera and choroid of humans and monkeys. *Investigative Ophthalmology & Visual Science*.

[28] Guymer R, Luthert P, Bird A (1999). Changes in Bruch’s membrane and related structures with age. *Progress in Retinal and Eye Research*.

[29] Ardeljan D, Chan CC (2013). Aging is not a disease: distinguishing age-related macular degeneration from aging. *Progress in Retinal and Eye Research*.

[30] Newsome DA, Huh W, Green WR (1987). Bruch’s membrane age-related changes vary by region. *Current Eye Research*.

[31] Okubo A, Rosa RH, Bunce CV (1999). The relationships of age changes in retinal pigment epithelium and Bruch’s membrane. *Investigative Ophthalmology & Visual Science*.

[32] Sheraidah G, Steinmetz R, Maguire J, Pauleikhoff D, Marshall J, Bird AC (1993). Correlation between lipids extracted from Bruch’s membrane and age. *Ophthalmology*.

[33] Curcio CA, Millican CL, Bailey T, Kruth HS (2001). Accumulation of cholesterol with age in human Bruch’s membrane. *Investigative Ophthalmology & Visual Science*.

[34] Ruberti JW, Curcio CA, Millican CL, Menco BPM, Huang J-D, Johnson M (2003). Quick-freeze/deep-etch visualization of age-related lipid accumulation in Bruch’s membrane. *Investigative Ophthalmology & Visual Science*.

[35] Bretillon L, Thuret G, Grégoire S (2008). Lipid and fatty acid profile of the retina, retinal pigment epithelium/choroid, and the lacrimal gland, and associations with adipose tissue fatty acids in human subjects. *Experimental Eye Research*.

[36] Karwatowski WSS, Jeffries TE, Duance VC, Albon J, Bailey AJ, Easty DL (1995). Preparation of Bruch’s membrane and analysis of the age-related changes in the structural collagens. *British Journal of Ophthalmology*.

[37] Guo L, Hussain AA, Limb GA, Marshall J (1999). Age-dependent variation in metalloproteinase activity of isolated human Bruch’s membrane and choroid. *Investigative Ophthalmology & Visual Science*.

[38] Hussain AA, Lee Y, Marshall J (2010). High molecular-weight gelatinase species of human Bruch’s membrane: compositional analyses and age-related changes. *Investigative Ophthalmology & Visual Science*.

[39] Kumar A, El-Osta A, Hussain AA, Marshall J (2010). Increased sequestration of matrix metalloproteinases in ageing human Bruch’s membrane: implications for ECM turnover. *Investigative Ophthalmology & Visual Science*.

[40] Kamei M, Hollyfield JG (1999). TIMP-3 in Bruch’s membrane: changes during aging and in age-related macular degeneration. *Investigative Ophthalmology & Visual Science*.

[41] Handa JT, Verzijl N, Matsunaga H (1999). Increase in the advanced glycation end product pentosidine in Bruch’s membrane with age. *Investigative Ophthalmology & Visual Science*.

[42] Glenn JV, Mahaffy H, Wu K (2009). Advanced glycation end product (AGE) accumulation on Bruch’s membrane: links to age-related RPE dysfunction. *Investigative Ophthalmology & Visual Science*.

[43] Murdaugh LS, Wang Z, Del Priore LV, Dillon J, Gaillard ER (2010). Age-related accumulation of 3-nitrotyrosine and nitro-A2E in human Bruch’s membrane. *Experimental Eye Research*.

[44] Ugarte M, Hussain AA, Marshall J (2006). An experimental study of the elastic properties of the human Bruch’s membrane-choroid complex: relevance to ageing. *British Journal of Ophthalmology*.

[45] Fisher RF (1987). The influence of age on some ocular basement membranes. *Eye*.

[46] Moore DJ, Hussain AA, Marshall J (1995). Age-related variation in the hydraulic conductivity of Bruch’s membrane. *Investigative Ophthalmology & Visual Science*.

[47] Starita C, Hussain AA, Marshall J (1995). Decreasing hydraulic conductivity of Bruch’s membrane: relevance to photoreceptor survival and lipofuscinoses. *The American Journal of Medical Genetics*.

[48] Starita C, Hussain AA, Pagliarini S, Marshall J (1996). Hydrodynamics of ageing Bruch’s membrane: implications for macular disease. *Experimental Eye Research*.

[49] Moore DJ, Clover GM (2001). The effect of age on the macromolecular permeability of human Bruch’s membrane. *Investigative Ophthalmology & Visual Science*.

[50] Hussain AA, Rowe L, Marshall J (2002). Age-related alterations in the diffusional transport of amino acids across the human Bruch’s-choroid complex. *Journal of the Optical Society of America A*.

[51] Hillenkamp J, Hussain AA, Jackson TL, Cunningham JR, Marshall J (2004). The influence of path length and matrix components on ageing characteristics of transport between the choroid and the outer retina. *Investigative Ophthalmology & Visual Science*.

[52] Hussain AA, Starita C, Hodgetts A, Marshall J (2010). Macromolecular diffusion characteristics of ageing human Bruch’s membrane: implications for age-related macular degeneration (AMD). *Experimental Eye Research*.

[53] Cai H, Del Priore L (2006). Bruch membrane aging alters the gene expression profile of human retinal pigment epithelium. *Current Eye Research*.

[54] Coffey AJH, Brownstein S (1986). The prevalence of macular drusen in postmortem eyes. *The American Journal of Ophthalmology*.

[55] Sarks SH, Arnold JJ, Killingsworth MC, Sarks JP (1999). Early drusen formation in the normal and aging eye and their relation to age related maculopathy: a clinicopathological study. *British Journal of Ophthalmology*.

[56] Lengyel I, Tufail A, Al Hosaini H, Luthert P, Bird AC, Jeffery G (2004). Association of drusen deposition with choroidal intercapillary pillars in the aging human eye. *Investigative Ophthalmology & Visual Science*.

[57] Bressler NM, Munoz B, Maguire MG (1995). Five-year incidence and disappearance of drusen and retinal pigment epithelial abnormalities. Waterman study. *Archives of Ophthalmology*.

[58] Sarks SH (1976). Ageing and degeneration in the macular region: a clinico pathological study. *British Journal of Ophthalmology*.

[59] Hoshino M, Mizuno K, Ichikawa H (1984). Aging alterations of retina and choroid of Japanese: light microscopic study of macular region of 176 eyes. *Japanese Journal of Ophthalmology*.

[60] Zealley B, De Grey AD (2013). Strategies for engineered negligible senescence. *Gerontology*.

[61] May CA (2005). Non-vascular smooth muscle cells in the human choroid: distribution, development and further characterization. *Journal of Anatomy*.

[62] Lütjen-Drecoll E (2006). Choroidal innervation in primate eyes. *Experimental Eye Research*.

[63] Booij JC, Baas DC, Beisekeeva J, Gorgels TGMF, Bergen AAB (2010). The dynamic nature of Bruch’s membrane. *Progress in Retinal and Eye Research*.

[64] Graebel WP, van Alphen GWHM (1977). The elasticity of sclera and choroid of the human eye, and its implications on scleral rigidity and accommodation. *Journal of Biomechanical Engineering*.

[65] Friberg TR, Lace JW (1988). A comparison of the elastic properties of human choroid and sclera. *Experimental Eye Research*.

[66] Chen K, Rowley AP, Weiland JD, Humayun MS (2013). Elastic properties of human posterior eye. *Journal of Biomedical Materials Research Part A*.

[67] Schroedl F, Brehmer A, Neuhuber WL, Kruse FE, May CA, Cursiefen C (2008). The normal human choroid is endowed with a significant number of lymphatic vessel endothelial hyaluronate receptor 1 (LYVE-1)-positive macrophages. *Investigative Ophthalmology & Visual Science*.

[68] Anderson OA, Jackson TL, Singh JK, Hussain AA, Marshall J (2008). Human transscleral albumin permeability and the effect of topographical location and donor age. *Investigative Ophthalmology & Visual Science*.

[69] Piguet B, Wells JA, Palmvang IB, Wormald R, Chisholm IH, Bird AC (1993). Age-related Bruch’s membrane change: a clinical study of the relative role of heredity and environment. *British Journal of Ophthalmology*.

[70] Samuelson DA, Lewis PA, MacKay E, Whitley RD (1999). The influence of aging and low zinc nutrition on the choroid in the pig: I. the melanocyte. *Veterinary Ophthalmology*.

[71] Wakamatsu K, Hu D-N, McCormick SA, Ito S (2008). Characterization of melanin in human iridal and choroidal melanocytes from eyes with various colored irides. *Pigment Cell & Melanoma Research*.

[72] Yaar M, Gilchrest BA (2001). Ageing and photoageing of keratinocytes and melanocytes. *Clinical and Experimental Dermatology*.

[73] Simon JD, Hong L, Peles DN (2008). Insights into melanosomes and melanin from some interesting spatial and temporal properties. *Journal of Physical Chemistry B*.

[74] Cherepanoff S, McMenamin P, Gillies MC, Kettle E, Sarks SH (2010). Bruch’s membrane and choroidal macrophages in early and advanced age-related macular degeneration. *British Journal of Ophthalmology*.

[75] May CA (1999). Mast cell heterogeneity in the human uvea. *Histochemistry and Cell Biology*.

[76] Gomez CR, Nomellini V, Faunce DE, Kovacs EJ (2008). Innate immunity and aging. *Experimental Gerontology*.

[77] Kvanta A (1995). Expression and regulation of vascular endothelial growth factor in choroidal fibroblasts. *Current Eye Research*.

[78] Li W, He Z, Li Y, Yanoff M (2000). Vascular endothelial growth factor regulates both apoptosis and angiogenesis of choriocapillaris endothelial cells. *Microvascular Research*.

[79] Browning AC, Dua HS, Amoaku WM (2008). The effects of growth factors on the proliferation and in vitro angiogenesis of human macular inner choroidal endothelial cells. *British Journal of Ophthalmology*.

